# Redetermination of conichalcite, CaCu(AsO_4_)(OH)

**DOI:** 10.1107/S1600536808024173

**Published:** 2008-08-06

**Authors:** Rachel R. Henderson, Hexiong Yang, Robert T. Downs, Robert A. Jenkins

**Affiliations:** a Department of Geosciences, University of Arizona, 1040 E. 4th Street, Tucson, AZ 85721-0077, USA

## Abstract

The crystal structure of conichalcite [calcium copper(II) arsenate(V) hydroxide], with ideal formula CaCu(AsO_4_)(OH), was redetermined from a natural twinned specimen found in the Maria Catalina mine (Chile). In contrast to the previous refinement from photographic data [Qurashi & Barnes (1963 ▶). *Can. Mineral.* **7**, 561–577], all atoms were refined with anisotropic displacement parameters and with the H atom located. Conichalcite belongs to the adelite mineral group. The Jahn–Teller-distorted [CuO_6_] octa­hedra share edges, forming chains running parallel to [010]. These chains are cross-linked by eight-coordinate Ca atoms and by sharing vertices with isolated AsO_4_ tetra­hedra. Of five calcium arsenate minerals in the adelite group, the [*M*O_6_] (*M* = Cu, Zn, Co, Ni and Mg) octa­hedron in conichalcite is the most distorted, and the donor–acceptor O—H⋯O distance is the shortest.

## Related literature

For background on the adelite mineral family, see: Qurashi & Barnes (1963 ▶, 1964 ▶); Qurashi *et al.* (1953 ▶). For structure refinements in the adelite group, see: Effenberger *et al.* (2002 ▶) for adelite, CaMgAsO_4_(OH); Clark *et al.* (1997 ▶) and Giuseppetti & Tadini (1988 ▶) for austinite, CaZnAsO_4_(OH); Yang *et al.* (2007 ▶) for cobaltaustinite, CaCoAsO_4_(OH); Cesbron *et al.* (1987 ▶) for nickelaustinite, CaNiAsO_4_(OH). Correlations between O—H streching frequencies and O—H⋯O donor–acceptor distances are given by Libowitzky (1999 ▶). Raman spectroscopic data on some minerals of the adelite group have been reported by Martens *et al.* (2003 ▶); for general background, see: Robinson *et al.* (1971 ▶).

## Experimental

### 

#### Crystal data

CaCu(AsO_4_)(OH)
*M* *_r_* = 259.57
Orthorhombic, 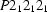

*a* = 7.3822 (2) Å
*b* = 5.8146 (2) Å
*c* = 9.2136 (3) Å
*V* = 395.49 (2) Å^3^
*Z* = 4
Mo *K*α radiation
μ = 15.03 mm^−1^
*T* = 293 (2) K
0.06 × 0.05 × 0.04 mm

#### Data collection

Bruker APEXII CCD diffractometer
Absorption correction: multi-scan (*TWINABS*; Sheldrick, 2008 ▶) *T*_min_ = 0.492, *T*_max_ = 0.585 (expected range = 0.461–0.548)
7088 measured reflections
1602 independent reflections
1487 reflections with *I* > 2σ(*I*)
*R*_int_ = 0.023

#### Refinement

*R*[*F* ^2^ > 2σ(*F* ^2^)] = 0.018
*wR*(*F* ^2^) = 0.038
*S* = 1.03
1602 reflections
79 parameters
All H-atom parameters refined
Δρ_max_ = 0.63 e Å^−3^
Δρ_min_ = −0.49 e Å^−3^
Absolute structure: Flack (1983 ▶), 644 Friedel pairs
Flack parameter: 0.00 (2)

### 

Data collection: *APEX2* (Bruker, 2003 ▶); cell refinement: *SAINT* (Bruker, 2005 ▶); data reduction: *SAINT*; program(s) used to solve structure: *SHELXS97* (Sheldrick, 2008 ▶); program(s) used to refine structure: *SHELXL97* (Sheldrick, 2008 ▶); molecular graphics: *XtalDraw* (Downs & Hall-Wallace, 2003 ▶); software used to prepare material for publication: *SHELXTL* (Sheldrick, 2008 ▶).

## Supplementary Material

Crystal structure: contains datablocks I, global. DOI: 10.1107/S1600536808024173/wm2185sup1.cif

Structure factors: contains datablocks I. DOI: 10.1107/S1600536808024173/wm2185Isup2.hkl

Additional supplementary materials:  crystallographic information; 3D view; checkCIF report

## Figures and Tables

**Table 1:** Selected bond lengths (Å)

Ca—O5^i^	2.3626 (13)
Ca—O3^ii^	2.3995 (17)
Ca—O4^iii^	2.4818 (16)
Ca—O2^iv^	2.5178 (17)
Ca—O4^v^	2.5281 (16)
Ca—O1^iv^	2.5462 (14)
Ca—O3	2.5786 (17)
Ca—O2	2.6264 (17)
Cu—O5	1.8850 (15)
Cu—O5^vi^	1.8855 (16)
Cu—O1^vi^	2.0666 (16)
Cu—O1	2.0688 (15)
Cu—O3^vii^	2.2976 (15)
Cu—O4^viii^	2.3882 (14)
As—O4	1.6749 (16)
As—O3	1.6779 (16)
As—O2	1.6796 (16)
As—O1	1.7099 (13)

Symmetry codes: (i) 
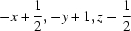
; (ii) 
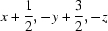
; (iii) 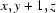; (iv) 
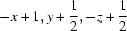
; (v) 
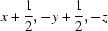
; (vi) 
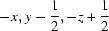
; (vii) 
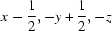
; (viii) 
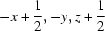
.

**Table 2:** Hydrogen-bond geometry (Å, °)

*D*—H⋯*A*	*D*—H	H⋯*A*	*D*⋯*A*	*D*—H⋯*A*
O5—H1⋯O2^ix^	0.86 (4)	1.91 (4)	2.678 (2)	149 (3)

Symmetry code: (ix) 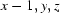.

**Fig. 1.**
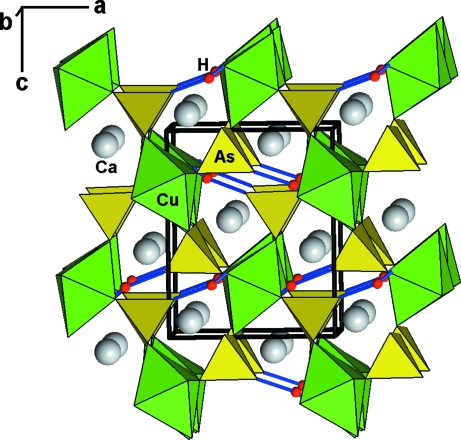
The crystal structure of conichalcite. Green octahedra, yellow tetrahedra, grey large sphares, and red small spheres represent [CuO6], [AsO4], Ca, and H, respectively. Hydrogen bonding is indicated with blue lines.

## supplementary crystallographic information

### Comment

Minerals of the adelite group crystallize with orthorhombic symmetry in space group *P*2_1_2_1_2_1_ (Qurashi & Barnes, 1963, 1964) and have a general chemical formula *A*^+,2+^*M*^2+,3+^(*X*^4+,5+,6+^O_4_)(OH), where *A* = Na, Ca, Pb, *M* = Al, Mg, Zn, Mn, Fe, Co, Cu, Ni, and *X* = Si, P, V, As. There are five calcium arsenates in this group: adelite CaMgAsO_4_(OH), austinite CaZnAsO_4_(OH), conichalcite CaCuAsO_4_(OH), nickelaustinite CaNiAsO_4_(OH), and cobaltaustinite, CaCoAsO_4_(OH). All structures of these calcium arsenate minerals have been determined previously (Qurashi & Barnes, 1963; Cesbron *et al.*, 1987; Giuseppetti & Tadini, 1988; Clark *et al.*, 1997; Effenberger *et al.*, 2002; Yang *et al.*, 2007). However, in our efforts to understand the relationships between the hydrogen bonding schemes and Raman spectra of hydrous minerals, we noted that the structural information of conichalcite needs to be improved, because this structure was refined by Qurashi & Barnes (1963) with X-ray intensity data collected by Qurashi *et al.* (1953) from precession photographs without anisotropic displacement parameters and localisation of the H atom position.

Conichalcite can be compared with the other Ca-arsenate minerals in the adelite group. The distorted [CuO_6_] octahedra (i.e. elongated tetragonal bipyramids) share edges to form chains running parallel to [010], which are cross-linked by Ca atoms and by sharing vertices with isolated AsO_4_ tetrahedra (Fig. 1). The principal difference among the five calcium arsenates in the group is manifested in the bonding environments around the octahedrally coordinated *M* cations. The average M—O bond lengths appear to decrease from <Zn—O> (= 2.106 Å) in austinite (Clark *et al.*, 1997), to <Cu—O> (= 2.099 Å) in conichalcite, <Co—O> (= 2.092 Å) in cobaltaustinite (Yang *et al.*, 2007), <Ni—O> (= 2.085 Å) in nickelaustinite (Cesbron *et al.*, 1987), and to <Mg—O> (= 2.075 Å) in adelite (Effenberger *et al.*, 2002). Of these [MO_6_] octahedra, the Cu-octahedron, due to its strong Jahn-Teller effect, displays the greatest distortion in terms of the tetragonal elongation and angle variance (Robinson *et al.*, 1971), which are 1.0229 and 23.58, respectively.

The donor-acceptor O5—H···O2 distance in conichalcite is 2.678 (2) Å, which is the shortest of all five Ca-arsenates in the adelite group [2.723 (2) Å in austinite (Clark *et al.*, 1997), 2.721 (7) Å in cobaltaustinite (Yang *et al.*, 2007), 2.73 (1) Å in nickelaustinite (Cesbron *et al.*, 1987), and 2.766 (2) Å in adelite (Effenberger *et al.*, 2002)]. As the O—H stretching frequencies (ν_OH_) increase with the O—H···O distance (Libowitzky,1999), we should expect the smallest ν_OH_ value for conichalcite and the largest for adelite among the five calcium arsenates in the adelite group. Indeed, the major ν_OH_ band positions determined from Raman spectra for conichalcite and adelite are, respectively, 3158 and 3550 cm^-1^ from Martens *et al.* (2003), or 3161 and 3423 cm^-1^ from the RRUFF project (http://rruff.info), with intermediate ν_OH_ values for the other three minerals (austinite, cobaltaustinite, and nickelaustinite).

### Experimental

The conichalcite crystal used in this study is from Maria Catalina mine, Pampa Larga Mining District, Tierra Amarilla, Chile, and is a sample from the RRUFF project (deposition No. R070430; http//rruff.info). The chemical composition, Ca(Cu_0.99_Zn_0.01_)(AsO_4_)(OH), was determined with a CAMECA SX50 electron microprobe (http//rruff.info).

### Refinement

The final refinement assumed a full occupancy of the metal site by Cu only, as the overall effects of the trace amount of Zn on the final structure results are negligible. In the final stages of the refinement it turned out that the measured crystal was racemically twinned with an approximate twin fraction of 4:1 (BASF = 0.21). The H atom was located from difference Fourier maps and its position was refined freely. The highest residual peak in is located 1.60 Å from the H atom, and the deepest hole is 0.63 Å from the Ca atom.

### Crystal data

**Table:**

CaCu(AsO_4_)(OH)	*F*_000_ = 492
*M**_r_* = 259.57	*D*_x_ = 4.359 Mg m^−^^3^
Orthorhombic, *P*2_1_2_1_2_1_	Mo *K*α radiation λ = 0.71073 Å
Hall symbol: P 2ac 2ab	Cell parameters from 3371 reflections
*a* = 7.3822 (2) Å	θ = 3.6–34.0º
*b* = 5.8146 (2) Å	µ = 15.03 mm^−^^1^
*c* = 9.2136 (3) Å	*T* = 293 (2) K
*V* = 395.49 (2) Å^3^	Euhedral, equant, green
*Z* = 4	0.06 × 0.05 × 0.04 mm

### Data collection

**Table:**

Bruker APEX2 CCD diffractometer	1602 independent reflections
Radiation source: fine-focus sealed tube	1487 reflections with *I* > 2σ(*I*)
Monochromator: graphite	*R*_int_ = 0.023
*T* = 293(2) K	θ_max_ = 34.0º
φ and ω scans	θ_min_ = 3.5º
Absorption correction: multi-scan(TWINABS; Sheldrick, 2008)	*h* = −9→11
*T*_min_ = 0.492, *T*_max_ = 0.585	*k* = −9→9
7088 measured reflections	*l* = −14→14

### Refinement

**Table:**

Refinement on *F*^2^	All H-atom parameters refined
Least-squares matrix: full	*w* = 1/[σ^2^(*F*_o_^2^) + (0.0151*P*)^2^ + 0.1227*P*] where *P* = (*F*_o_^2^ + 2*F*_c_^2^)/3
*R*[*F*^2^ > 2σ(*F*^2^)] = 0.018	(Δ/σ)_max_ < 0.001
*wR*(*F*^2^) = 0.038	Δρ_max_ = 0.63 e Å^−^^3^
*S* = 1.03	Δρ_min_ = −0.49 e Å^−^^3^
1602 reflections	Extinction correction: SHELXL97 (Sheldrick, 2008), Fc^*^=kFc[1+0.001xFc^2^λ^3^/sin(2θ)]^-1/4^
79 parameters	Extinction coefficient: 0.0029 (5)
Primary atom site location: structure-invariant direct methods	Absolute structure: Flack (1983), 644 Friedel pairs
Secondary atom site location: difference Fourier map	Flack parameter: 0.00 (2)
Hydrogen site location: difference Fourier map	

### Special details

**Table:**

Geometry. All e.s.d.'s (except the e.s.d. in the dihedral angle between two l.s. planes) are estimated using the full covariance matrix. The cell e.s.d.'s are taken into account individually in the estimation of e.s.d.'s in distances, angles and torsion angles; correlations between e.s.d.'s in cell parameters are only used when they are defined by crystal symmetry. An approximate (isotropic) treatment of cell e.s.d.'s is used for estimating e.s.d.'s involving l.s. planes.
Refinement. Refinement of *F*^2^ against ALL reflections. The weighted *R*-factor *wR* and goodness of fit *S* are based on *F*^2^, conventional *R*-factors *R* are based on *F*, with *F* set to zero for negative *F*^2^. The threshold expression of *F*^2^ > σ(*F*^2^) is used only for calculating *R*-factors(gt) *etc*. and is not relevant to the choice of reflections for refinement. *R*-factors based on *F*^2^ are statistically about twice as large as those based on *F*, and *R*- factors based on ALL data will be even larger.

### Fractional atomic coordinates and isotropic or equivalent isotropic displacement parameters (Å^2^)

**Table:**

	*x*	*y*	*z*	*U*_iso_*/*U*_eq_	
Ca	0.61727 (5)	0.72961 (8)	0.07340 (4)	0.01133 (12)	
Cu	−0.00416 (4)	−0.00002 (6)	0.25029 (4)	0.00898 (8)	
As	0.36728 (2)	0.26438 (4)	0.08118 (2)	0.00768 (6)	
O1	0.18844 (17)	0.2450 (3)	0.19847 (15)	0.0135 (3)	
O2	0.5395 (2)	0.3313 (3)	0.19256 (18)	0.0187 (4)	
O3	0.3514 (2)	0.4927 (3)	−0.02947 (17)	0.0157 (3)	
O4	0.3885 (2)	0.0147 (3)	−0.00782 (15)	0.0145 (4)	
O5	−0.13880 (18)	0.2539 (3)	0.31777 (14)	0.0113 (3)	
H1	−0.229 (5)	0.232 (6)	0.260 (3)	0.055 (11)*	

### Atomic displacement parameters (Å^2^)

**Table:**

	*U*^11^	*U*^22^	*U*^33^	*U*^12^	*U*^13^	*U*^23^
Ca	0.01169 (18)	0.0119 (2)	0.01041 (18)	−0.00064 (17)	−0.00072 (13)	0.00020 (18)
Cu	0.00885 (11)	0.00621 (12)	0.01190 (12)	0.00029 (9)	0.00211 (8)	−0.00121 (8)
As	0.00801 (8)	0.00659 (9)	0.00845 (9)	0.00006 (8)	0.00059 (7)	0.00014 (9)
O1	0.0141 (6)	0.0107 (7)	0.0157 (6)	−0.0025 (7)	0.0035 (5)	−0.0006 (7)
O2	0.0146 (7)	0.0212 (9)	0.0205 (8)	−0.0029 (6)	−0.0048 (6)	0.0004 (7)
O3	0.0201 (7)	0.0107 (7)	0.0164 (7)	0.0011 (7)	0.0044 (7)	0.0035 (6)
O4	0.0181 (8)	0.0096 (7)	0.0158 (8)	0.0018 (6)	0.0022 (7)	−0.0018 (6)
O5	0.0106 (5)	0.0102 (6)	0.0132 (6)	0.0000 (8)	0.0002 (5)	−0.0003 (6)

### Geometric parameters (Å, °)

**Table:**

Ca—O5^i^	2.3626 (13)	Cu—O5^vi^	1.8855 (16)
Ca—O3^ii^	2.3995 (17)	Cu—O1^vi^	2.0666 (16)
Ca—O4^iii^	2.4818 (16)	Cu—O1	2.0688 (15)
Ca—O2^iv^	2.5178 (17)	Cu—O3^vii^	2.2976 (15)
Ca—O4^v^	2.5281 (16)	Cu—O4^viii^	2.3882 (14)
Ca—O1^iv^	2.5462 (14)	As—O4	1.6749 (16)
Ca—O3	2.5786 (17)	As—O3	1.6779 (16)
Ca—O2	2.6264 (17)	As—O2	1.6796 (16)
Cu—O5	1.8850 (15)	As—O1	1.7099 (13)
			
O5^i^—Ca—O3^ii^	75.88 (5)	O4^v^—Ca—O2	77.15 (5)
O5^i^—Ca—O4^iii^	73.62 (5)	O1^iv^—Ca—O2	79.00 (5)
O3^ii^—Ca—O4^iii^	89.42 (5)	O3—Ca—O2	61.06 (5)
O5^i^—Ca—O2^iv^	151.07 (5)	O5—Cu—O5^vi^	177.68 (6)
O3^ii^—Ca—O2^iv^	108.51 (6)	O5—Cu—O1^vi^	98.01 (6)
O4^iii^—Ca—O2^iv^	77.79 (5)	O5^vi^—Cu—O1^vi^	84.26 (6)
O5^i^—Ca—O4^v^	74.41 (5)	O5—Cu—O1	84.21 (6)
O3^ii^—Ca—O4^v^	76.55 (5)	O5^vi^—Cu—O1	93.52 (6)
O4^iii^—Ca—O4^v^	147.36 (3)	O1^vi^—Cu—O1	177.66 (7)
O2^iv^—Ca—O4^v^	134.48 (5)	O5—Cu—O3^vii^	91.88 (6)
O5^i^—Ca—O1^iv^	141.59 (5)	O5^vi^—Cu—O3^vii^	88.82 (6)
O3^ii^—Ca—O1^iv^	73.13 (5)	O1^vi^—Cu—O3^vii^	84.84 (6)
O4^iii^—Ca—O1^iv^	127.50 (5)	O1—Cu—O3^vii^	95.85 (6)
O2^iv^—Ca—O1^iv^	62.85 (5)	O5—Cu—O4^viii^	84.76 (6)
O4^v^—Ca—O1^iv^	76.76 (5)	O5^vi^—Cu—O4^viii^	94.77 (6)
O5^i^—Ca—O3	72.94 (5)	O1^vi^—Cu—O4^viii^	89.81 (6)
O3^ii^—Ca—O3	147.75 (2)	O1—Cu—O4^viii^	89.67 (5)
O4^iii^—Ca—O3	74.21 (5)	O3^vii^—Cu—O4^viii^	173.23 (6)
O2^iv^—Ca—O3	95.19 (5)	O4—As—O3	113.27 (7)
O4^v^—Ca—O3	102.41 (5)	O4—As—O2	115.43 (8)
O1^iv^—Ca—O3	138.68 (5)	O3—As—O2	103.94 (8)
O5^i^—Ca—O2	117.86 (6)	O4—As—O1	108.94 (8)
O3^ii^—Ca—O2	145.22 (5)	O3—As—O1	112.45 (8)
O4^iii^—Ca—O2	124.48 (5)	O2—As—O1	102.33 (7)
O2^iv^—Ca—O2	75.44 (3)		

Symmetry codes: (i) −*x*+1/2, −*y*+1, *z*−1/2; (ii) *x*+1/2, −*y*+3/2, −*z*; (iii) *x*, *y*+1, *z*; (iv) −*x*+1, *y*+1/2, −*z*+1/2; (v) *x*+1/2, −*y*+1/2, −*z*; (vi) −*x*, *y*−1/2, −*z*+1/2; (vii) *x*−1/2, −*y*+1/2, −*z*; (viii) −*x*+1/2, −*y*, *z*+1/2.

### Hydrogen-bond geometry (Å, °)

**Table:**

*D*—H···*A*	*D*—H	H···*A*	*D*···*A*	*D*—H···*A*
O5—H1···O2^ix^	0.86 (4)	1.91 (4)	2.678 (2)	149 (3)

Symmetry codes: (ix) *x*−1, *y*, *z*.
